# A238 BILE ACID COMPOSITION AND DIETARY FAT: IMPLICATIONS FOR CROHN’S DISEASE IN A COHORT OF HEALTHY FIRST-DEGREE RELATIVES

**DOI:** 10.1093/jcag/gwac036.238

**Published:** 2023-03-07

**Authors:** A Neustaeter, J Shao, M Xue, C Antonio Hernández Rocha, S -H Lee, H Leibovitzh, K Madsen, J B Meddings, O Espin-Garcia, A M Griffiths, P Moayyedi, A H Steinhart, R Panancionne, H Huynh, K Jacobson, G Aumais, D Mack, C Bernstein, J K Marshall, W Xu, W Turpin, K Croitoru

**Affiliations:** 1 University of Toronto, Toronto; 2 University of Alberta, Edmonton; 3 Cumming School of Medicine, Calgary; 4 The Hospital for Sick Children,, Toronto; 5 McMaster University, Hamilton; 6 University of Calgary; 7 University of Alberta, Calgary; 8 University of British Columbia, Vancouver; 9 Montreal University, Montreal; 10 University of Ottawa, Ottawa; 11 University of Manitoba, Winnipeg, Canada

## Abstract

**Background:**

Crohn’s disease (CD) is a chronic relapsing inflammatory disease of the gastrointestinal tract. The etiology of CD may arise from complex interactions including host genetics, diet, and the intestinal microbiome. Increased consumption of saturated fats, characteristic of the Western diet, is a known risk factor for CD. Dietary fat (DF) is absorbed by the host through the release of primary bile acids (PBAs) and bio-transformed by the microbiome into secondary bile acids (SBAs). Altogether, bile acids (BAs) can act as signaling molecules involved in host immune regulation and potentially in CD onset.

**Purpose:**

To investigate the relationship between CD risk, BAs, and DF, and evaluate the predictive performance of CD onset of these factors by developing machine learning models.

**Method:**

We used samples healthy first-degree relatives (FDRs) recruited as part of the Crohn’s Colitis Canada- Genes, Environment, Microbial (GEM) project. Those who developed CD (n=87) were matched 1:4 by age, sex, follow-up time, and geographic location with control FDRs remaining healthy (n=347).

Serum, urine, and stool BA were measured using ultrahigh Performance Liquid Chromatography-Tandem Mass Spectroscopy. DF types were derived from food frequency questionnaire data. We used conditional logistic regressions to identify associations between CD onset, BAs (n=93), and DFs (n=9). We further explored the relationships of significant CD-related BAs and DF via Generalized Estimation Equations. Finally, we used a tree-based machine-learning algorithm (XGBoost) with 5-fold cross-validation to assess the prediction performance of CD onset using BA from all sources as well as DF. Two-sided p<0.05 was considered significant.

**Result(s):**

In total, 10 of 93 BAs, and two of nine DFs were significantly associated with increased odds of CD onset (p<0.05). Additionally, five BAs were significantly associated with DF (p<0.05). Serum-derived BAs had the best predictive performance for CD, with a mean AUC of 0.70 [95% CI: 0.63;0.76], followed by stool derived BAs with a mean AUC= 0.65 [0.55;0.75], and followed by urine derived Bas with a mean AUC= 0.57 [0.48;0.66]. Lastly DF was not a predictive marker of CD onset with a mean AUC= 0.50 [0.41;0.60].

**Conclusion(s):**

This study suggests that BAs are associated with the pathogenesis of CD and the effects may be influenced by DF. Serum-derived BAs may be able to better predict the risk of CD than other stool or urine derived BA, while DF is not directly implicated in CD risk.

Submitted on behalf of the CCC-GEM consortium.

**Funding**

Crohn’s and Colitis Canada Genetics Environment Microbial (CCC-GEM) III

The Leona M. and Harry B. Helmsley Charitable Trust

Kenneth Croitoru is the recipient of the Canada Research Chair in Inflammatory Bowel Diseases

The International Organization for the Study of Inflammatory Bowel Diseases (IOIBD)

Jingcheng Shao is the recipient of a Data Science Institute Summer Undergraduate Data Science award

**Disclosure of Interest:**

None Declared

